# Carbon nanomaterial-derived lung burden analysis using UV-Vis spectrophotometry and proteinase K digestion

**DOI:** 10.1186/s12989-020-00377-9

**Published:** 2020-09-11

**Authors:** Dong-Keun Lee, Soyeon Jeon, Jiyoung Jeong, Kyung Seuk Song, Wan-Seob Cho

**Affiliations:** 1 Lab of Toxicology, Department of Health Sciences, Dong-A University, 37, Nakdong-daero 550 beon-gil, Saha-gu, Busan, 49315 Republic of Korea; 2 Korea Conformity Laboratories, 8, Gaetbeol-ro 145 beon-gil, Yeonsu-gu, 21999 Incheon, Republic of Korea

**Keywords:** Carbon black, Nanodiamond, Multi-walled carbon nanotube, Carbon nanofiber, Graphene, Lung burden

## Abstract

**Background:**

The quantification of nanomaterials accumulated in various organs is crucial in studying their toxicity and toxicokinetics. However, some types of nanomaterials, including carbon nanomaterials (CNMs), are difficult to quantify in a biological matrix. Therefore, developing improved methodologies for quantification of CNMs in vital organs is instrumental in their continued modification and application.

**Results:**

In this study, carbon black, nanodiamond, multi-walled carbon nanotube, carbon nanofiber, and graphene nanoplatelet were assembled and used as a panel of CNMs. All CNMs showed significant absorbance at 750 nm, while their bio-components showed minimal absorbance at this wavelength. Quantification of CNMs using their absorbance at 750 nm was shown to have more than 94% accuracy in all of the studied materials. Incubating proteinase K (PK) for 2 days with a mixture of lung tissue homogenates and CNMs showed an average recovery rate over 90%. The utility of this method was confirmed in a murine pharyngeal aspiration model using CNMs at 30 μg/mouse.

**Conclusions:**

We developed an improved lung burden assay for CNMs with an accuracy > 94% and a recovery rate > 90% using PK digestion and UV-Vis spectrophotometry. This method can be applied to any nanomaterial with sufficient absorbance in the near-infrared band and can differentiate nanomaterials from elements in the body, as well as the soluble fraction of the nanomaterial. Furthermore, a combination of PK digestion and other instrumental analysis specific to the nanomaterial can be applied to organ burden analysis.

## Background

Inhalation is the most common and hazardous route of exposure to nanomaterials in an occupational setting. Inhalation of nanomaterials produces a higher deposition rate of the micron-sized particles within the alveoli as a result of their size-dependent aerodynamic properties [1–3]. Furthermore, deposited particles exhibit limited clearance rates from the alveoli due to the absence of mucociliary clearance. The clearance of these nanomaterials from the alveoli is influenced by the physicochemical properties of the material including size, shape, functionalization, and dissolution [4–6]. Because of the long retention period for nanomaterials in the lungs, the Organization for Economic Cooperation and Development (OECD) testing guidelines call for repeated inhalation studies (i.e., TG 412 and 413) and were revised in 2018 to include lung burden measurements showing lung clearance kinetics for the material of interest [7, 8].

There are various methods which can be used to measure the lung burden of non-labelled nanomaterials. Generally, lung burden analysis can be divided into two steps: (1) collection of nanomaterials from the lung and (2) quantification of nanomaterials using instrumental analysis. In the first step, chemical or enzymatic digestion methods are commonly used to collect nanomaterials from the lung tissue. Chemical digestion methods using acids, alkalis, and oxidants are all common but chemical digestion reagents can damage the structure of the nanomaterials resulting in defects, dissolution and oxidation [9]. Enzymatic digestion uses proteinase or collagenase with a chemical lysis buffer and has been proposed as an alternative to chemical lysis, as this degradation approach seems to limit structural damage of the nanomaterials [9, 10]. In the second step, nanomaterials can be measured by various instrumental analyses including inductively coupled plasma mass spectrometry (ICP-MS), fluorometry, and optical absorbance spectrometry. For carbon nanomaterials (CNMs), the determination of the concentration is challenging because of the difficulty of measuring carbon in an organic matrix. Several approaches have been used to measure CNMs in biological matrices, including gel electrophoresis [11], programmed thermal analysis (PTA) [9], Raman spectroscopy [12], and near-infrared (NIR) spectroscopy [13]. However, there are calls for the development of more efficient and reliable measurement methods or protocols for CNMs in an organ.

Carbon nanomaterials (CNMs) such as carbon nanotubes, graphene and carbon black are considered hazardous materials when inhaled because of their bio-persistence, high bio-durability, and unique physicochemical properties including their size and shape [14–17]. Therefore, the precise evaluation of the kinetics of CNMs is required for proper hazard and risk assessment of CNMs. In this study, we developed an efficient and reliable protocol for measuring the lung burden of various CNMs including carbon black (CB), nanodiamond (ND), multi-walled carbon nanotube (MWCNT), carbon nanofiber (CNF), and graphene nanoplatelet (GNP) using proteinase K (PK) tissue digestion and quantification of the recovered CNMs using a UV-Vis spectrophotometer.

## Results

### Working scheme

**Fig. 1:**
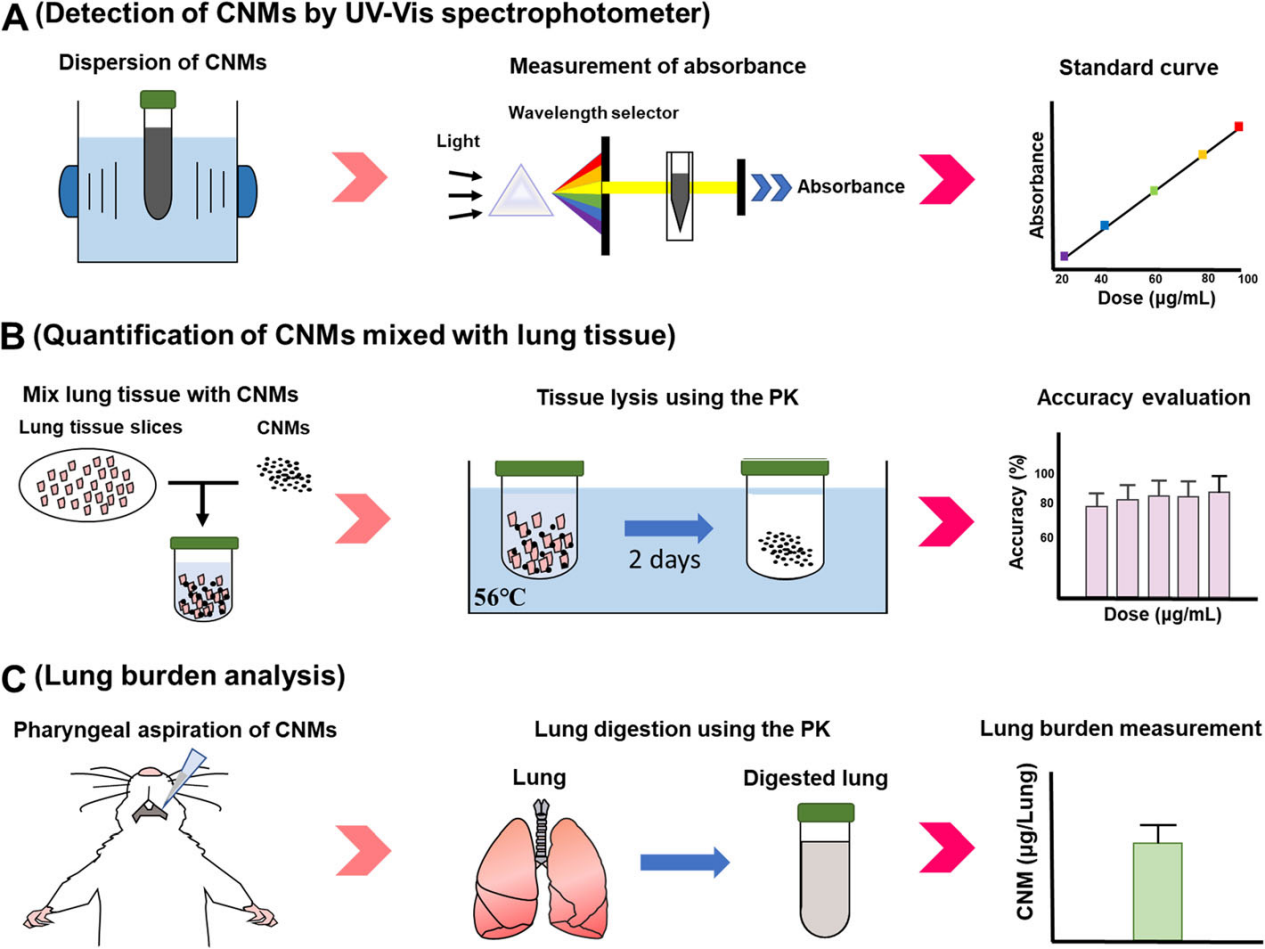
Schematic workflow for lung burden analysis. **a**, Selection of the optimal wavelength to quantify CNM concentration without interference from the bio-components of the tissue homogenate. **b** Quantification of CNM concentration after mixing with lung tissue homogenates. In this step, proteinase K (PK) digestion was used to collect CNMs from lung tissue homogenates. **c**, in vivo evaluation of this lung burden assay using a murine pharyngeal aspiration model

Figure 1 is a schematic of the workflow used to evaluate CNM lung burden in this study. Five types of CNM including CB, ND, MWCNT, CNF, and GNP were selected as test materials, which allowed us to cover most of the available CNMs currently employed in research and industries. The first step in our assay development was to identify the optimal wavelength for measuring CNM concentration. This wavelength is needed to reduce any interference from the biocomponents of the lung homogenates while still giving accurate CNM quantitation (Fig. 1a). The second step was evaluating the quantification of CNMs after they were added to the lung tissue homogenates (Fig. 1b). To do this CNMs were collected from lung tissue homogenates following PK digestion. Finally, we needed to evaluate this assay in an in vivo model; here we measured CNM lung burden at 24 h post pharyngeal aspiration in mice (Fig. 1c).

### Transmission electron microscopy (TEM) analysis of CNMs

**Fig. 2:**
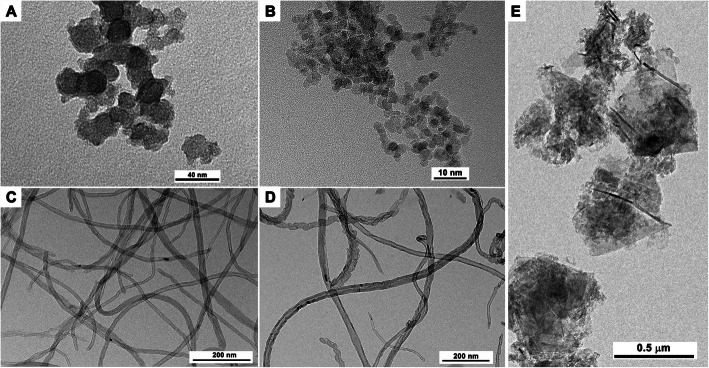
The shape and morphology of various CNMs evaluated using transmission electron microscopy. **a**, carbon black; **b**, nanodiamonds; **c**, carbon nanotube; **d**, carbon nanofiber; **e**, graphene nanoplatelet

Representative TEM images of the CNMs are presented in Fig. 2. CB and ND were spherical with an average size of 14 ± 0.2 nm and 4.87 ± 0.4 nm, respectively. MWCNT and CNF were tubular. The size, specific surface area, and IG/ID ratio of CNMs are presented in Table 1. The diameter and length of MWCNT were 16.7 ± 0.2 nm and 3.55 μm, respectively. The diameter and length of CNF were 24.79 ± 0.4 nm and < 10 μm, respectively. GNP was plate-shaped with a mean diameter of 512 ± 9.7 nm. The BET specific surface area of CNMs was ranged 184–500 m^2^/g. The ID/IG ratio of CNMs showed variable values by the types of CNMs.

**Table 1:** The physicochemical properties of CNMs

CNMs	Size	Surface area (m^2^/g)	Raman (ID/IG)
CB	14 ± 0.2 nm	254	0.35
ND	4.87 ± 0.4 nm	279	0.3
MWCNT	Diameter: 16.7 ± 0.2 nm Length: 3. 55 μm	224	1.46
CNF	Diameter: 24.79 ± 0.4 nm Length: < 10 μm	184	1.02
GNP	512 ± 9.7 nm	500	1.43

### Optimal wavelength selection for measuring CNM concentration with minimal interference

**Fig. 3:**
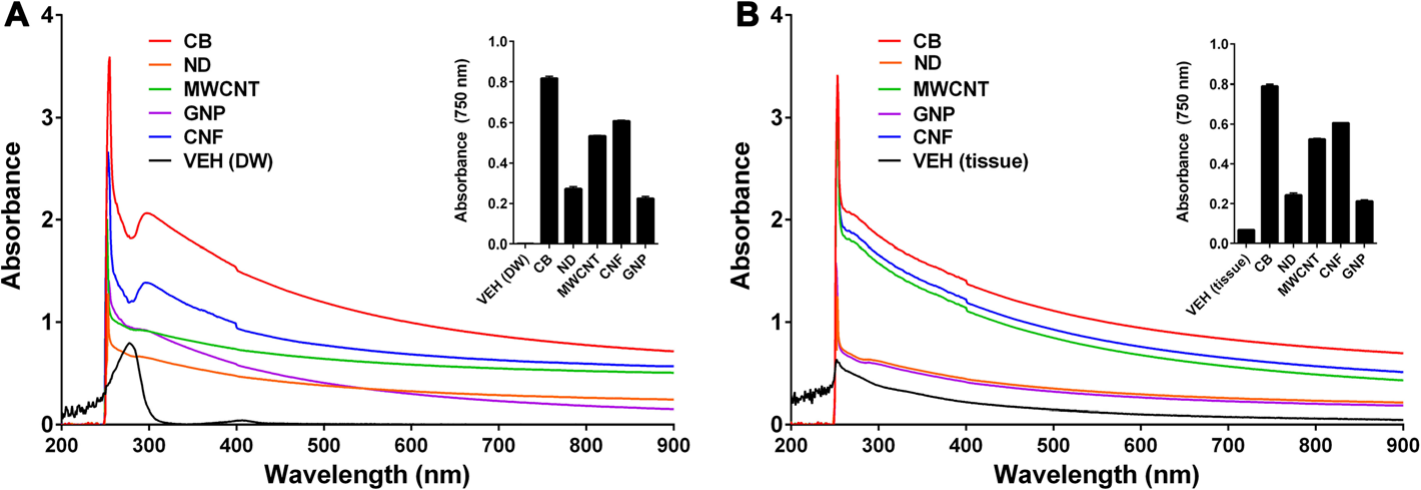
The selection of an optimal wavelength to quantify CNMs without interference from tissue homogenates. **a**, Absorbance of CNMs or vehicle control [VEH (DW), DW with 3% FBS]. **b**, Absorbance of vehicle control [VEH (tissue), tissue lysates in DW] and CNMs collected from a mixture of 25 μg/mL CNMs and 0.02 g (dry weight) lung tissue homogenates following treatment with 200 μg of proteinase K (PK). The insert figure represents that the absorbance of the bio-components was lower at 750 nm after treatment with PK, making it the optimal wavelength to quantify CNMs without interference from the tissue homogenate

CNMs dispersed in distilled water (DW) with 3% foetal bovine serum (FBS) were shown to have increased absorbance in the 200–300 nm range, which then reached a plateau and stabilised until absorbance reached 900 nm (Fig. 3a). The absorbance of the empty vehicle (DW with 3% FBS) used in this study also showed increasing absorbance between 200 and 300 nm, but its absorbance was reduced to nearly zero after 500 nm. In addition, CNMs/lung homogenate mixtures exhibited slightly higher absorbance values from 200 to 900 nm when compared to CNMs in DW with 3% FBS (Fig. 3b). However, the absorbance of the lung tissue lysis solution was shown to be reduced after 750 nm (approximately 0.068), which meant that this wavelength could be used to successfully measure CNMs in mixed biological solutions without interference. CNM at 25 μg/mL were shown to have an absorbance of 0.219 at 750 nm (Fig. 3b), confirming that 750 nm was the optimal wavelength for evaluations of CNM concentration in lung tissue homogenates. Because this technique uses optical absorbance in the near-infrared region, any nanomaterials having a strong absorbance in this range could be quantified using a similar approach.

### Quantification of CNMs dispersed in DW with 3% FBS

**Fig. 4:**
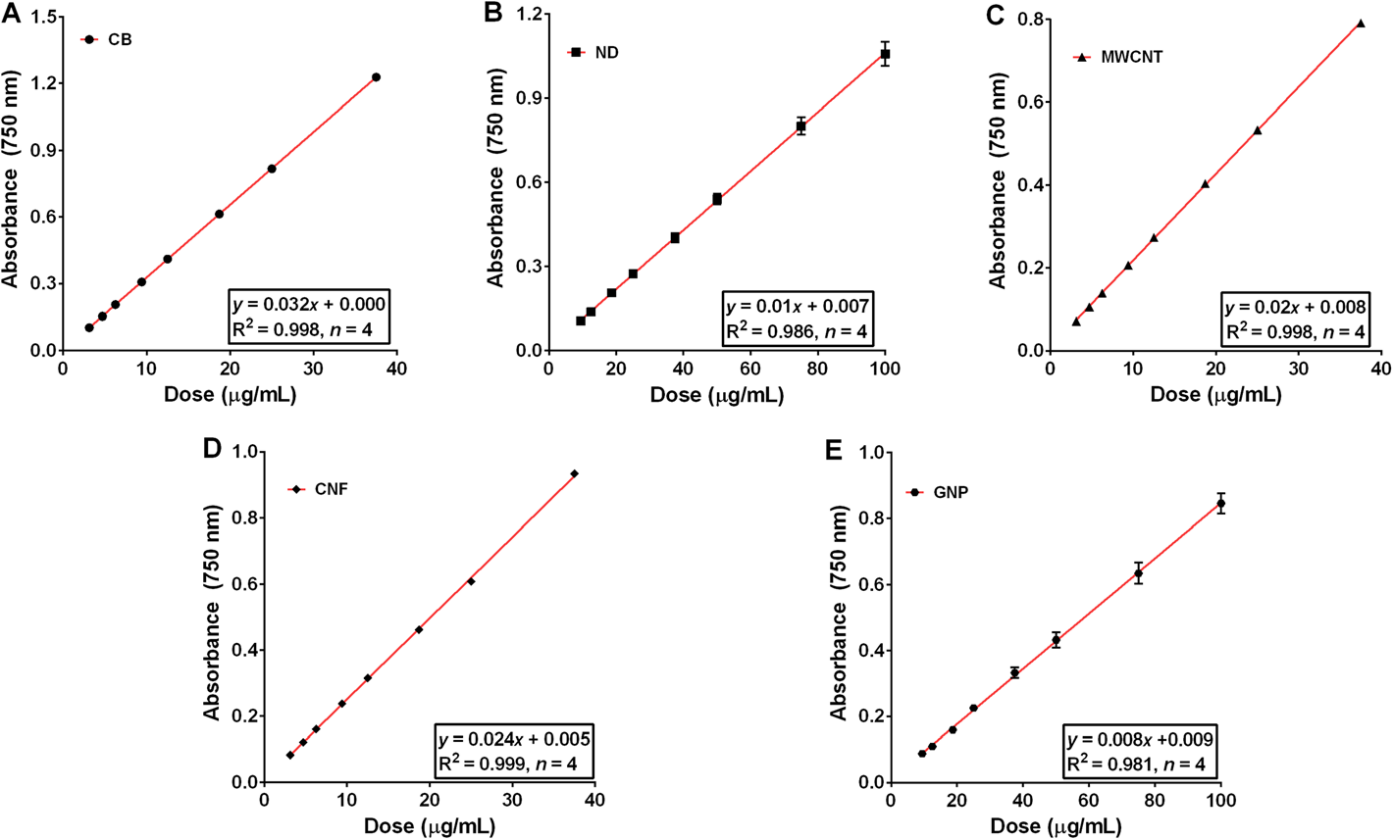
The standard curve fit of CNMs using a UV-Vis spectrophotometer. CNMs were dispersed in distilled water with 3% foetal bovine serum and absorbance was measured at 750 nm. **a**, carbon black (CB); **b**, nanodiamond (ND); **c**, multi-walled carbon nanotube (MWCNT); **d**, carbon nanofiber (CNF); **e**, graphene nanoplatelet (GNP). Data are expressed as mean ± SEM and *n* = 4

To evaluate the detection limit for CNMs, a range of CNM concentrations (0 to 1000 μg/mL) were resuspended in DW with 3% FBS and then subjected to evaluation at 750 nm. The lower and upper detection limits for a linear dose-response were 0.39–50 μg/mL for CB, MWCNT, and CNF, and 1.56–200 μg/mL for ND and GNP (Fig. S1, see Supporting Information). To evaluate the accuracy and reproducibility of this detection method, four concentrations of each of the CNMs (i.e., 10, 20, 30, and 300 μg/mL) were tested. The R^2^ values of standard curve fits for all CNMs were more than 0.98 (Fig. 4). The detection accuracy (%) for all CNMs was more than 94% compared to the target concentration (Table 2).

**Table 2:** The accuracy (%) of the UV-Vis spectrophotometer technique when measuring CNM concentration

CNMs	Target concentration (μg/mL)			
	10	20	30	300^a^
CB				
Measured	9.53 ± 0.28	19.37 ± 0.66	28.76 ± 0.47	285.49 ± 1.04
Accuracy %	95.3 ± 2.84	96.8 ± 3.33	95.8 ± 1.57	95.1 ± 0.34
ND				
Measured	9.51 ± 0.26	19.07 ± 0.76	28.52 ± 1.03	286.06 ± 4.05
Accuracy %	95.1 ± 2.61	95.3 ± 3.82	95.0 ± 3.43	95.3 ± 1.35
MWCNT				
Measured	9.69 ± 0.18	19.29 ± 0.57	28.75 ± 1.11	285.12 ± 4.55
Accuracy %	96.9 ± 1.84	96.4 ± 2.85	95.8 ± 3.70	95.0 ± 1.51
CNF				
Measured	9.42 ± 0.56	19.03 ± 0.57	28.69 ± 1.06	286.04 ± 2.22
Accuracy %	94.2 ± 5.67	95.1 ± 2.88	95.6 ± 3.55	95.3 ± 0.74
GNP				
Measured	9.63 ± 0.11	19.48 ± 0.87	29.22 ± 1.10	289.88 ± 9.92
Accuracy %	96.2 ± 1.12	97.4 ± 4.37	97.4 ± 3.68	96.6 ± 3.30

The accuracy (%) was calculated by comparison with the target concentration weighed during sample preparation

^a^The target concentrations were selected as 10, 20, and 30 μg/mL for non-diluted samples and 300 μg/mL for samples needed dilution

The data are presented as mean ± SEM from four independent measurements

### Quantification of CNMs from the lung tissue homogenates

**Fig. 5:**
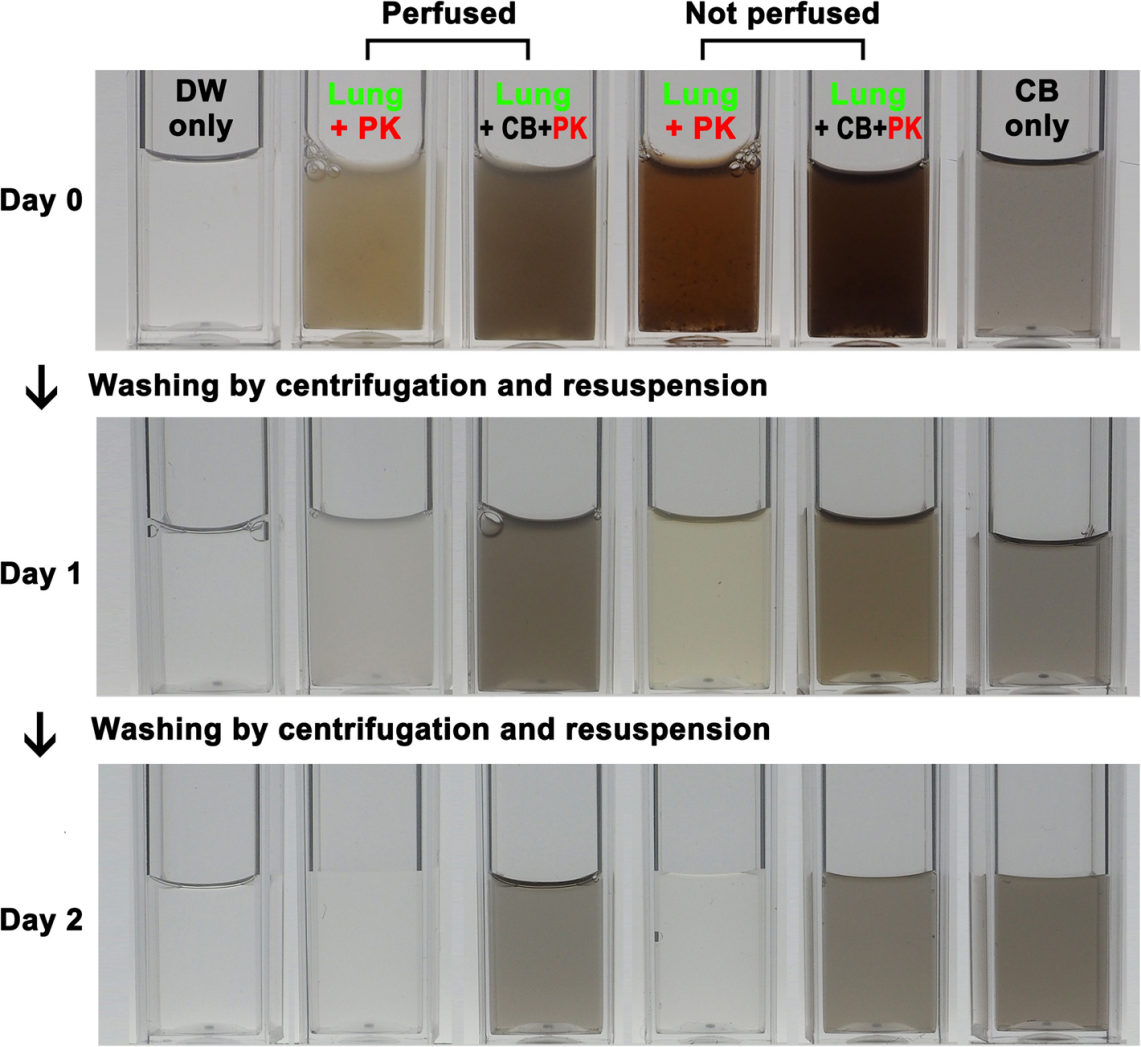
The visual changes in the lung tissue homogenates after incubation with proteinase K (PK). Carbon black (CB) was selected as a representative CNM and a mixture of CB at 25 μg and lung tissue homogenates at 0.02 g (dry weight) were incubated in 1 mL of Tris buffer (pH 8.0) containing 200 μg PK. After 24 h samples were washed by centrifugation and incubated for another 24 h in 1 mL of Tris buffer (pH 8.0) containing 200 μg PK. Note that CB could be completely recovered from the lung tissue homogenates following PK digestion and centrifugation. The perfusion step did not influence nanomaterial recovery

**Fig. 6:**
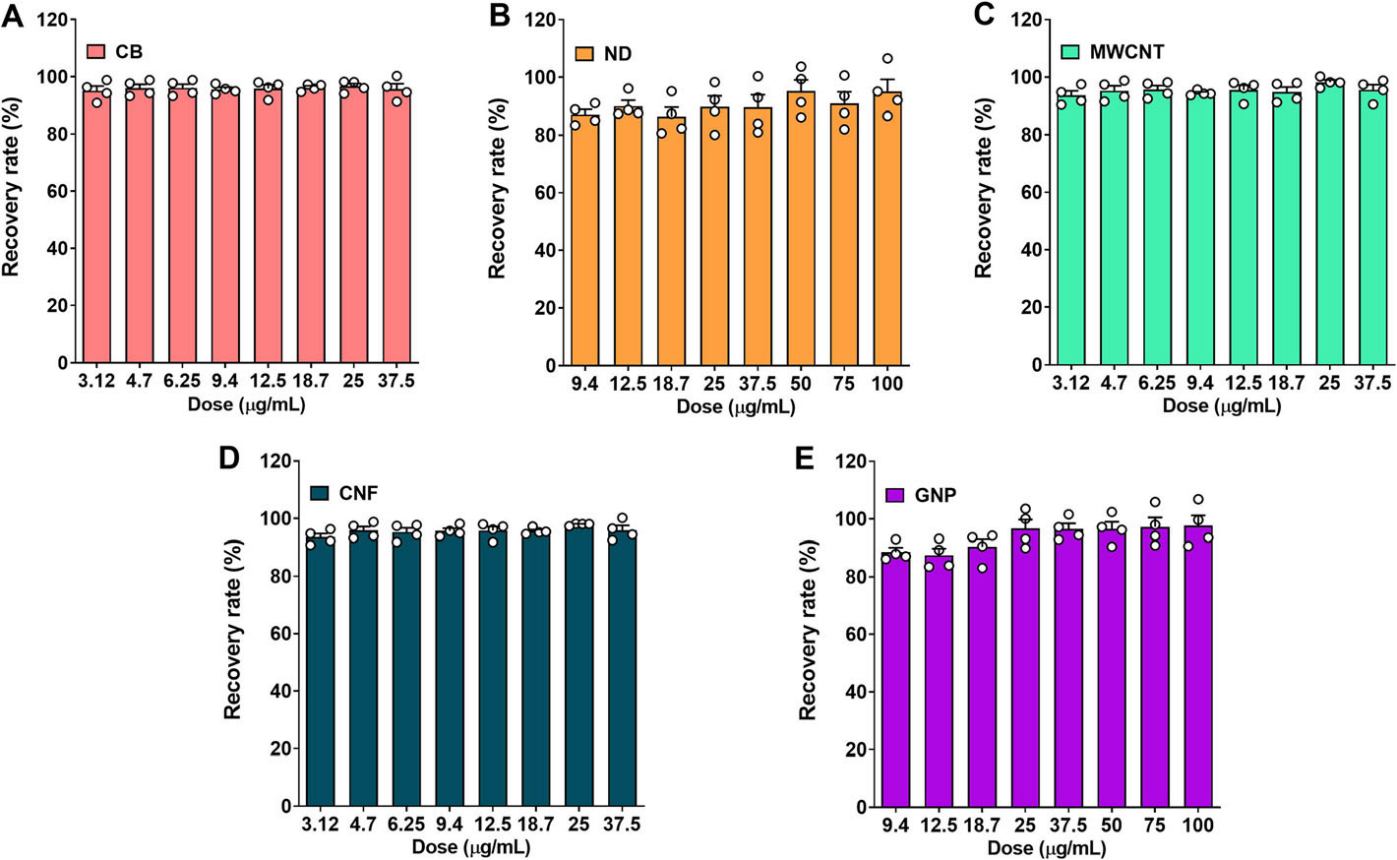
The recovery rate of CNMs mixed with lung tissue homogenates following proteinase K (PK) digestion. All CNMs in the tested dose ranges showed a more than 86% recovery rate from lung tissue homogenates following PK digestion. **a**, carbon black; **b**, nanodiamond; **c**, carbon nanotube; **d**, carbon nanofiber; **e**, graphene nanoplatelet. Data are expressed as mean ± SEM and *n* = 4. The detailed numeric data are presented in Tables S1 and S2 (see Supporting Information)

The second step in developing our lung burden assay was to evaluate its efficacy in a tissue setting. To do this, CNMs were mixed with lung tissue homogenates, treated with PK and then evaluated using the UV-Vis spectrophotometer technique described above. First 0.02 g (dry weight) of lung tissue homogenates were treated with 1 mL of Tris buffer (pH 8.0) containing 200 μg PK at 56 °C and showed complete lysis within 2 days (Fig. 5). The presence of erythrocytes did not influence the efficacy of the PK digestion as lung tissues were completely digested regardless of perfusion (Fig. 5). Thus, the main experiment was performed with lung tissues without perfusion. All CNMs were properly detected using this technique and the recovery percentage for the CNMs between 3.1 to 100 μg/mL was over 86% and mean recovery percentage of tested concentrations was over 90% for all types of CNMs (Fig. 6 and Tables S1 and S2, see Supporting Information). It is worth noting that the UV-Vis spectrophotometer can detect both higher and lower CNM concentrations than the ones used here supporting its widespread utility in this type of application. The loss rate of CNMs by mechanical processes such as washing and centrifugation was ranged about 3–7% (Table 3). Because the loss rate was dependent on the concentration of samples, it was slightly increased as increasing the concentration.

**Table 3:** The loss rate (%) of CNMs by mechanical processes such as washing and centrifugation

Nominal Dose (μg/mL)	10	20	30
CB	4.60 ± 0.78^a^	4.84 ± 1.14	4.60 ± 0.68
ND	4.19 ± 1.06	3.95 ± 1.27	3.87 ± 0.44
MWCNT	6.20 ± 0.71	5.28 ± 0.94	4.54 ± 0.79
CNF	5.73 ± 1.70	4.25 ± 0.99	4.11 ± 0.75
GNP	4.97 ± 1.15	4.81 ± 1.14	4.26 ± 1.91

^a^The recovered concentration was divided by the initial dose to calculate the loss rate

Data are mean ± SEM and *n* = 4 for each concentration

### Lung burden analysis after pharyngeal aspiration of CNMs in mice

**Fig. 7:**
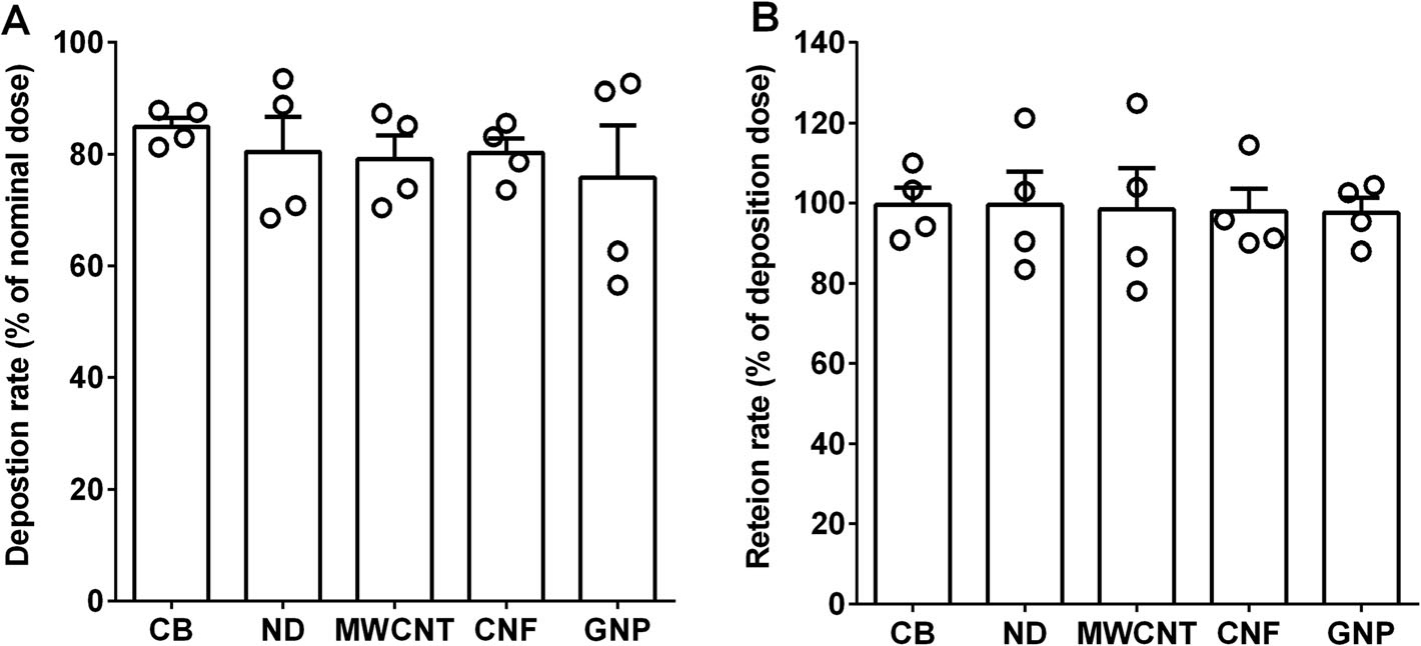
A pilot study to evaluate the lung deposition rate (**a**) and retention rate (**b**) of CNMs. To evaluate the deposition rate in comparison with the nominal treatment dose, mice were sacrificed immediately after a single pharyngeal aspiration and lung burden analysis was performed. To evaluate the retention rate of CNMs at 24 h, mice were sacrificed at 24 h after a pharyngeal aspiration. Mice were given 30 μg/mouse of CNMs and the concentration of these CNMs in the lungs was quantified using PK digestion and UV-Vis spectrophotometry at each time-point. Data are expressed as mean ± SEM and *n* = 4

As a pilot study, we evaluated the deposition rate at time zero and retention rate at 24 h after a single pharyngeal aspiration of CNMs. The deposition rates of CB, ND, MWCNT, CNF, and GNP at time zero compared to the nominal treatment dose (30 μg/mouse) were 84.9, 80.4, 79.1, 80.2, and 75.7%, respectively (Fig. 7a). While, the retention rates of CB, ND, MWCNT, CNF, and GNP at 24 h after aspiration compared to that of time zero were 99.5, 99.5, 98.4, 97.9, and 97.5%, respectively (Fig. 7b).

## Discussion

Measuring the lung burden of nanomaterials is now mandatory under the revised inhalation toxicity testing guidelines (i.e., TG412 and TG413) published by the OECD [7, 8], and there is an ongoing project of testing guidelines for toxicokinetics to accommodate nanomaterials [18]. In addition, new or improved methodologies to evaluate the concentration of nanomaterials in biological samples is essential for continued research including evaluating nanomaterials in biomedical applications. Thus, this study was designed to create a novel methodology to quantify CNMs deposited in the lung using PK enzymatic digestion and UV-Vis spectrophotometry.

The recovery of CNMs from organ tissue homogenates is critical for the success of organ burden assays and various chemical cocktails or enzymes have been suggested for the digestion of these organ tissues [9]. Chemical cocktails to digest organ tissues use oxidants, acids, and alkalis. These cocktails are hazardous to human health and some chemicals like nitric acid can induce defects or degradation of nanomaterials even in their most stable formats [19]. Because CNMs are commonly quantified using thermal or optical methods structural defects or degradation of CNMs can result in inaccurate quantification. In addition, most organ burden analysis of metal-based nanomaterials use acid digestions to lyse the organ tissues and nanomaterials [20, 21]. However, this method cannot discriminate between nanomaterial-derived metal ions and tissue-derived metals or bio-persistent nanomaterials from dissolved ions [22, 23]. For example, the iron concentration collected by acid digestion of organs treated with iron oxide nanomaterials can be derived from iron in the organ, iron from dissolved iron oxide in the body fluid such as lysosomal fluid, or iron from bio-persistent iron oxide. Thus, the extraction of nanomaterials from the organ without defect or degradation is critical for the accurate quantification of nanomaterials in organ tissues. The enzymatic digestion of these tissues could therefore provide a solution to these problems. Here, we showed that enzymatic digestion could facilitate the recovery of nanomaterials from tissue homogenate without damaging the CNMs and allowed for the evaluation of morphological changes like defects or biotransformation [24].

Mineralization of CNMs is required for the acid digestion methods such as HCl, nitric acid, and hydrofluoric acid. However, some of the chemical digestion agents such as Solvable (PerkinElmer, Waltham, MA, USA) and Clean 99-K200 (Clean Chemical, Osaka, Japan) and enzymatic digestion methods do not require the mineralization process [9, 25]. Because the mineralization process can reduce the recovery of CNMs, the wet process such as proteinase-K can have advantages to collect CNMs in the biological matrices. To our knowledge, this the first report demonstrating the recovery of CNMs from the mixture of tissue homogenates and nanomaterials using the PK digestion method. Here, we were able to recover about 90% of the CNMs mixed with lung tissue homogenates. The loss of CNMs during the lung burden analysis using PK digestion method can be due to the mechanical loss (e.g., 3–7%) such as washing and centrifugation and inaccuracy (e.g., < 6%) of the UV-Vis spectrophotometer technique as shown in this study. Furthermore, PK enzymatic digestion was shown to work perfectly with or without perfusion, which is advantageous for toxicity studies.

After collecting nanomaterials from the organ tissue, it is important to select an appropriate method for instrumental analysis; this will allow proper quantification of the relevant material. CNMs are generally measured using programmed thermal analysis (PTA) like NIOSH 5040 [5, 26]. However, PTA analysis requires expensive instrumentation and inaccuracies can occur when CNMs are damaged during the extraction process. This method shows only about 50% recovery in lung burden analyses from a 28-day inhalation study of MWCNT [5, 9]. Here, we have been able to show that UV-Vis spectrophotometry can accurately measure five types of CNM over a range of concentrations (0.39–50 μg/mL for CB, MWCNT, and CNF; 1.56–200 μg/mL for ND and GNP) with more than 94% accuracy. Although this study showed a detection limit of MWCNT as 0.39 μg/mL, a previous study suggests that the limit of detection for MWCNT using a UV-Vis spectrophotometer is 0.025 μg/mL [10], which is more sensitive than any of the metal-labelling methods like Ni-labelling, which showed a 0.1 μg/mL limit of detection [27], and PTA, which showed 0.2 μg limit of detection [9]. In comparison with this study, the lower detection limit of MWCNT performed by Zhang et al. [10] could be due to the difference in the physicochemical properties such as length, diameter, and dispersibility [28–30]. Furthermore, the use of UV-Vis spectrophotometer is available to other types of nanomaterials such as metal oxides (Table S3, see Supporting Information).

## Conclusions

In this study, we developed an optimized lung burden assay with over 90% accuracy and 83% recovery rates designed to evaluate CNMs. This methodology relies on a PK digestion and UV-Vis spectrophotometry. This method can also be applied to other nanomaterials with significant absorbance in the near-infrared band. In addition, this technique can be applied to differentiate between the nanomaterials from the elements of the body or soluble fraction. Furthermore, the combination of PK digestion and other instrumental analysis, such as PTA, ICP-MS, fluorometer, or particle counter, could help to overcome the limitations in quantifying other nanomaterials in biological samples.

## Materials and methods

### Selection of CNMs and TEM analysis

A panel of CNMs was assembled to include various types of nanomaterials including CB, ND, MWCNT, CNF, and GNP. Based on the morphology, two materials can be classified as particles (i.e., CB and ND), two as fibers (i.e., MWCNT and CNF), and one as a platelet (i.e., GNP). All CNMs were provided from commercial sources: CB (# Printex 90; Evonik Degussa GmbH, Frankfurt, Germany), ND (# ND1; S.W. Chemicals Co., Ltd., Gunsan, Korea), MWCNT (#CM-100; Hanwha Nanotech Co., Seoul, Korea), CNF (# T-CNF; Carbon Nano-material Technology Co., Pohang, Korea), and graphene (# 06–0230; Stream Chemical Inc., Newburyport, MA, USA). The size and shape of the CNMs were evaluated by TEM (JEM-1200EXII, JEOL, Tokyo, Japan) as described in our previous study [31]. The size of the CNMs was calculated by counting at least 300 CNMs using a built-in analysis program (JEOL). Raman spectroscopy was used on the CNMs to evaluate defects using a WITec alpha300 system (WITec GmbH, Ulm, Germany) with incident laser light at a wavelength of 532 nm. The surface area of the CNMs was measured with the Brunauer–Emmett–Teller method using a BELSORP-mini II (BEL Japan Inc.).

### Dispersion of CNMs

The degree of dispersion of the preparations is critical because NIR signals are known to be dispersion-dependent [29, 30]. Because of the hydrophobic nature of the CNMs, serum was used as the dispersion medium to provide a protein corona, which ensured proper dispersion of the CNMs within the media [32]. Furthermore, a water bath sonicator was applied to breakup agglomerates, which was broadly used in the process of nanomaterial dispersion [30, 33, 34]. Briefly, CNM powders were dispersed in DW containing 30% *v/v* heat-inactivated FBS and sonicated using a bath sonicator (Saehan Sonic, Seoul, Korea) to break up agglomerates. The operation condition of a bath sonicator was 40 kHz frequency and 400 W output power. Then, DW was added to make up the final working solution of CNMs, and the concentration of FBS was kept to less than 3% *v/v*. The target concentrations of the stock solution and working solution to evaluate the dispersion of CNM was 1 mg/mL and 25 μg/mL, respectively. The dispersibility of stock solution was measured by optical absorbance at 750 nm after diluting with DW at 25 μg/mL. After the selected optimal sonication duration of stock solution, the working solution was prepared at 25 μg/mL in DW and the dispersibility was evaluated after further sonication for 10–30 min. Because each CNMs needs a nanomaterial-specific duration of sonication for the best dispersion efficacy, the stock solution and working solution were sonicated with different durations (Table 4 and Fig. S2). All CNMs were stable up to 4 h with some minor variations between types of CNMs (Table 4 and Fig. S3). All standards and samples were measured within 10 min after the dispersion process.

**Table 4:** Duration of sonication for the best dispersion of CNMs

CNMs	CB	ND	MWCNT	CNF	GNP
Duration of sonication					
Stock	80 min	10 min	80 min	80 min	30 min
Working	10 min	10 min	10 min	10 min	10 min
Duration of the dispersion stability					
Working	4 h	16 h	4 h	4 h	4 h

### Measurement of CNMs dispersed in DW using a UV-Vis spectrophotometer

To evaluate the accuracy CNM concentration measurements on the UV-Vis spectrophotometer, the absorbance spectra of well-dispersed CNMs in 3% FBS DW were measured at 200–900 nm in quartz cuvettes using a UV-Vis spectrophotometer (Lambda 365, Perkin-Elmer, Waltham, Massachusetts, USA). Based on these results and those from several previous studies [35–37], we selected an absorption wavelength of 750 nm for all of the CNMs experiments. To estimate the linear dosage range for the standard curve various concentrations of CNMs from 0 to 1000 μg/mL were tested evaluated using the spectrophotometer, a standard curve of 8 concentrations (3.1, 4.7, 6.2, 9.4, 12.5, 18.7, 25, and 37.5 μg/mL for CB, MWCNT, and CNF; 9.4, 12.5, 18.7, 25, 37.5, 50, 75, and 100 μg/mL for ND and GNP) was selected for further experiments. To evaluate the accuracy and reproducibility, we selected target concentrations as 10, 20, and 30 μg/mL for non-diluted samples and 300 μg/mL for samples needed dilution. These target concentrations were not included in the data points used to calculate the calibration regression. Four independent measurements were performed for each to evaluate the accuracy and reproducibility of this system of measurement.

### Recovery of CNMs from lung tissue homogenates

Six-week-old specific-pathogen-free female ICR mice were purchased from Samtako (Gyeonggi-do, Korea). The mice were maintained and handled in accordance with the procedures approved by the Institutional Animal Care and Use Committee of Dong-A University. Animals were acclimatized for one week prior to experimentation then they were anaesthetized with isoflurane (Piramal Critical Care, Bethlehem, PA, USA) using a VetEquip rodent anaesthesia system (Pleasanton, CA, USA) and sacrificed by exsanguination via the interior vena cava. Then, the lung was perfused via the right ventricle with pre-warmed PBS containing 3.8% sodium citrate solution (Sigma-Aldrich, St. Louis, MO, USA). The perfused and non-perfused lungs were cut into pieces and dried in an oven at 60 °C for 48 h and crushed using a tissue homogenizer (Thomas Scientific, Swedesboro, NJ, USA). A total of 0,02 g (dry weight) of lung tissue homogenate was mixed with the CNM mixture. Then, 1 mL of PK digestion buffer [50 mM Tris-HCl, 10 mM CaCl_2_, and 200 μg PK (Promega, Madison, WI, USA), pH 8.0] was added and incubated for 24 h at 56 °C. Samples were then centrifuged at 15000×*g* for 20 min and the supernatant was removed. The pellets were resuspended in 1 mL of PK digestion buffer and sonicated for 5 min in a bath sonicator (Saehan Sonic) and incubated at 56 °C for a further 24 h. These suspensions were centrifuged at 15000×*g* for 20 min and the pellets were resuspended in 1 mL of DW and sonicated for 5 min in a bath sonicator (Saehan Sonic). The recovered CNMs were quantified using the UV-Vis spectrophotometer as described above.

### Evaluation of the loss rate of CNMs during the lung burden analysis

We evaluated the mechanical loss rate of CNMs during various processes in the lung burden analysis such as washing and centrifugation. Before starting the experiment, the concentration of dispersed CNMs at nominal concentrations of 10, 20, and 30 μg/mL were measured by UV-Vis spectrophotometer. Then, the suspensions of CNMs were processed with the identical procedures described in “Recovery of CNMs from lung tissue homogenates” without the addition of lung tissue homogenates. We excluded lung tissue homogenates to exclude the possible interference by the bio-components in the UV-Vis spectrophotometer technique and to focus on the mechanical loss rate of CNMs such as washing and centrifugation. Then, the recovered concentrations of CNMs were expressed as loss percentage compared to the initial concentration.

### Lung burden analysis after a single pharyngeal aspiration in mice

**Fig. 8:**
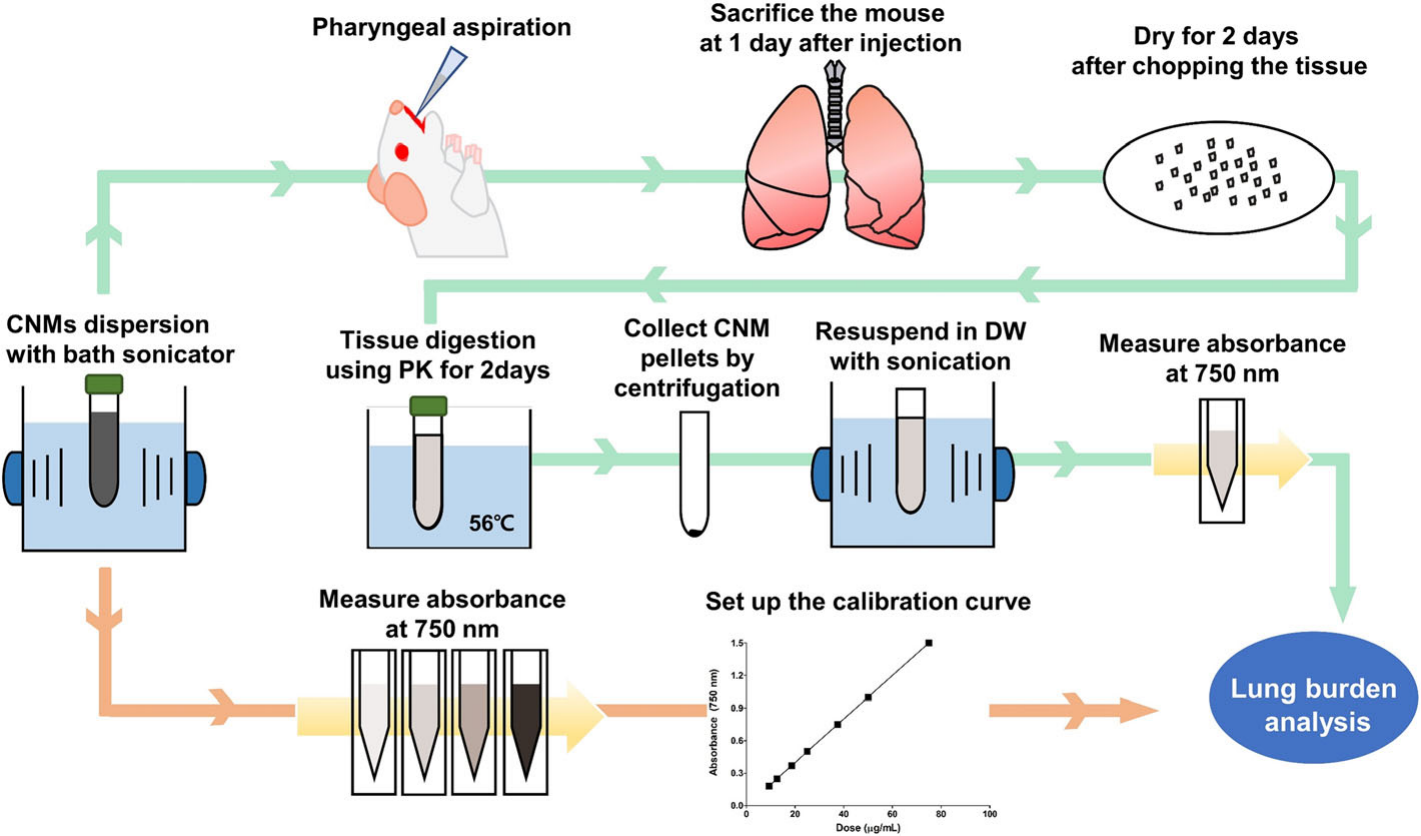
Schematic workflow of the pilot study for establishing the lung burden of CNMs in vivo

A pilot study of lung burden analysis was performed after a single pharyngeal aspiration of CNMs in mice. A schematic of the workflow for this study is presented in Fig. 8. Six-week-old female ICR mice (Samtako) were acclimatized for one week prior to experimentation. To perform the pharyngeal aspiration, mice were anaesthetized with isoflurane (Piramal Critical Care) and placed on a board in a near-vertical position. Then, a suspensions of well-dispersed CNMs in PBS with 3% (*v/v*) heat-inactivated mouse serum was loaded into the mouth and aspirated by holding the tongue at full extension and covering the nose. The aspiration volume was 50 μL/mouse and 3% mouse serum in PBS served as the vehicle control. The treatment dose was 30 μg/mouse. To evaluate the deposition rate in comparison with the nominal treatment dose, mice were sacrificed immediately after a single pharyngeal aspiration and lung burden analysis was performed. The deposition rate was calculated by dividing the lung burden at time zero with the nominal treatment dose. To evaluate the retention rate of CNMs at 24 h, mice were sacrificed at 24 h after a pharyngeal aspiration. The retention rate was calculated by dividing the lung burden at 24 h with that of time zero. At each time-point, mice were sacrificed by removing blood from the inferior vena cava under deep isoflurane anaesthesia. Lung tissue was cut into pieces and dried in an oven at 60 °C for 48 h. Dried lung tissues were weighed and crushed using a tissue homogenizer (Thomas Scientific). Then, 1 mL of PK digestion buffer containing 200 μg PK was added to 0.02 g (dry weight) lung homogenate and incubated for 24 h at 56 °C. Samples were centrifuged at 15000×*g* for 20 min to collect pellets containing CNMs and undigested lung tissue, and then resuspended in 1 mL of PK digestion buffer and incubated further 24 h at 56 °C. Finally, these samples were then sonicated for 5 min. The CNMs were collected via centrifugation at 15000×*g* for 20 min and resuspended in DW with 5 min sonication. The recovered CNMs were quantified using the UV-Vis spectrophotometer as described above.

### Statistical analysis

The data are presented as mean ± SEM and linear regression was applied to the standard curve fit. GraphPad Prism software (ver. 6.0; La Jolla, CA) was used to draw the graphs and perform all the statistical analysis.

## Supplementary information

**Additional file 1: Figure S1.** Measurement of the concentration of CNMs using a UV-Vis spectrophotometer. CNMs were dispersed in distilled water with 3% FBS and tested up to 1000 μg/mL. The absorbance was measured at 750 nm wavelength. Note that the lower and upper detection limits for a linear dose-response were 0.39–50 μg/mL for CB, MWCNT, and CNF, and 1.56–200 μg/mL for ND and GNP. (A), carbon black; (B), nanodiamond; (C), multi-walled carbon nanotube; (D), carbon nanofiber; (E), graphene nanoplatelet. **Figure S2**. Evaluation of the dispersibility of CNMs. The time-course dispersibility of stock solution (A) and working solution (B) of CNMs. (A), To evaluate the dispersibility of the stock solution, 1 mg/mL stock solution was sonicated for 10 min – 100 min. Then, at each time-point, the stock solution was diluted in DW at 25 μg/mL with vigorous vortexing for 30 s and measured the optical density at 750 nm. (B), To evaluate the duration of sonication for working solution, the working solution (25 μg/mL) of each NM after an optimal sonication duration of stock solution (see Table 4) was sonicated further up to 30 min and optical density was measured at 750 nm. *n* = 4. **Figure S3**. Duration of the dispersion stability of CNMs. The working solution of CNMs at 25 μg/mL was sonicated for 10 min after an optimal sonication duration of stock solution (see Table 4). Then, the duration of the dispersion stability was measured at each time-point up to 24 h. *n* = 4. **Table S1**. The recovery rates of CB, MWCNT, and CNF from lung tissue homogenates following proteinase K digestion with quantification using the UV-Vis spectrophotometer technique. **Table S2**. The recovery rates of ND and GNP from lung tissue homogenates following proteinase K digestion with quantification using the UV-Vis spectrophotometer technique. **Table S3**. The screening result of NIR absorbance at 750 nm of various types of nanomaterials.

## Data Availability

The datasets used and/or analyzed during the current study are available from the corresponding author on reasonable request.

## Abbreviations

CB: Carbon black
CNF: Carbon nanofiber
CNMs: Carbon nanomaterials
MWCNT: Multi-walled carbon nanotube
DW: Distilled water
GNP: Graphene nanoplatelet
ICP-MS: Inductively coupled plasma mass spectrometry
ND: Nanodiamond
PBS: Phosphate-buffered saline
PK: Proteinase K
PTA: Programmed thermal analysis
SEM: Standard error of the mean
TEM: Transmission electron microscopy
