# Supplementary material for: Carbon nanomaterial-derived lung burden analysis using UV-Vis spectrophotometry and proteinase K digestion
Source: Part Fibre Toxicol. 2020 Sep 11;17:43. doi: 10.1186/s12989-020-00377-9 (PMC7488454; doi:10.1186/s12989-020-00377-9)
Supplement: Supplementary file 1 — Additional file 1: Figure S1. Measurement of the concentration of CNMs using a UV-Vis spectrophotometer. CNMs were dispersed in distilled water with 3% FBS and tested up to 1000 μg/mL. The absorbance was measured at 750 nm wavelength. Note that the lower and upper detection limits for a linear dose-response were 0.39–50 μg/mL for CB, MWCNT, and CNF, and 1.56–200 μg/mL for ND and GNP. (A), carbon black; (B), nanodiamond; (C), multi-walled carbon nanotube; (D), carbon nanofiber; (E), graphene nanoplatelet. Figure S2. Evaluation of the dispersibility of CNMs. The time-course dispersibility of stock solution (A) and working solution (B) of CNMs. (A), To evaluate the dispersibility of the stock solution, 1 mg/mL stock solution was sonicated for 10 min – 100 min. Then, at each time-point, the stock solution was diluted in DW at 25 μg/mL with vigorous vortexing for 30 s and measured the optical density at 750 nm. (B), To evaluate the duration of sonication for working solution, the working solution (25 μg/mL) of each NM after an optimal sonication duration of stock solution (see Table 4) was sonicated further up to 30 min and optical density was measured at 750 nm. n = 4. Figure S3. Duration of the dispersion stability of CNMs. The working solution of CNMs at 25 μg/mL was sonicated for 10 min after an optimal sonication duration of stock solution (see Table 4). Then, the duration of the dispersion stability was measured at each time-point up to 24 h. n = 4. Table S1. The recovery rates of CB, MWCNT, and CNF from lung tissue homogenates following proteinase K digestion with quantification using the UV-Vis spectrophotometer technique. Table S2. The recovery rates of ND and GNP from lung tissue homogenates following proteinase K digestion with quantification using the UV-Vis spectrophotometer technique. Table S3. The screening result of NIR absorbance at 750 nm of various types of nanomaterials. [file 12989_2020_377_MOESM1_ESM.docx]

Supporting Information for

Carbon nanomaterial-derived lung burden analysis using UV-Vis spectrophotometry and proteinase K digestion

Dong-Keun Lee^1^, Soyeon Jeon^1^, Jiyoung Jeong^1^, Kyung Seuk Song^2^, Wan-Seob Cho^1^

^1^Lab of Toxicology, Department of Health Sciences, Dong-A University, 37, Nakdong-daero 550 beon-gil, Saha-gu, Busan 49315, Republic of Korea

^2^Korea Conformity Laboratories, 8, Gaetbeol-ro 145 beon-gil, Yeonsu-gu, 21999, Incheon, Republic of Korea

^*^Correspondence author:

Professor Wan-Seob Cho

Lab of Toxicology, Department of Medicinal Biotechnology

College of Health Sciences, Dong-A University, 37, Nakdong-daero, 550 beon-gil, Busan, 49315, Republic of Korea

Tel: +82-51-200-7563, E-mail: wcho@dau.ac.kr

**Figure S1.** Measurement of the concentration of CNMs using a UV-Vis spectrophotometer. CNMs were dispersed in distilled water with 3% FBS and tested up to 1000 μg/mL. The absorbance was measured at 750 nm wavelength. Note that the lower and upper detection limits for a linear dose-response were 0.39 – 50 µg/mL for CB, MWCNT, and CNF, and 1.56 – 200 µg/mL for ND and GNP. (A), carbon black; (B), nanodiamond; (C), multi-walled carbon nanotube; (D), carbon nanofiber; (E), graphene nanoplatelet.


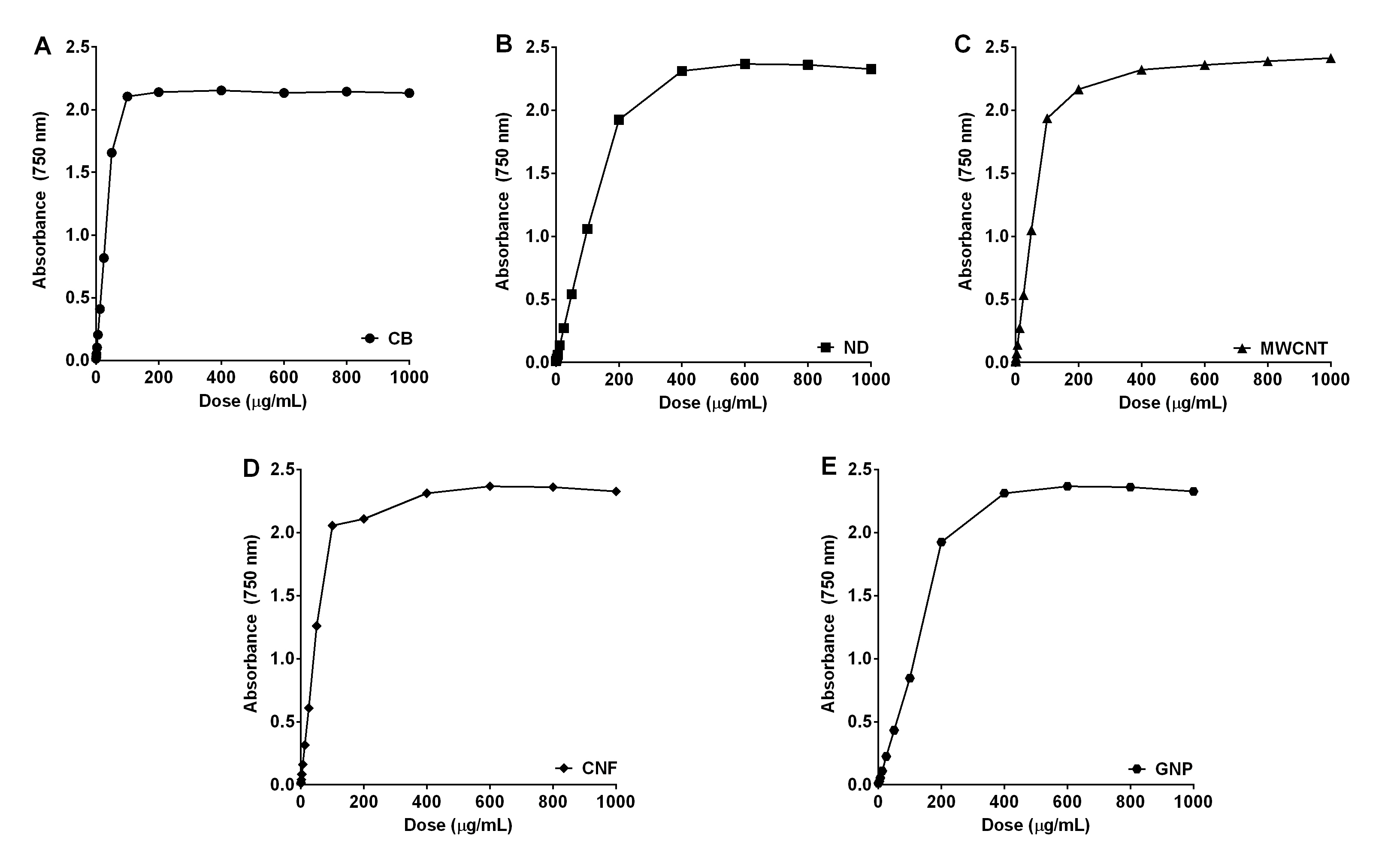

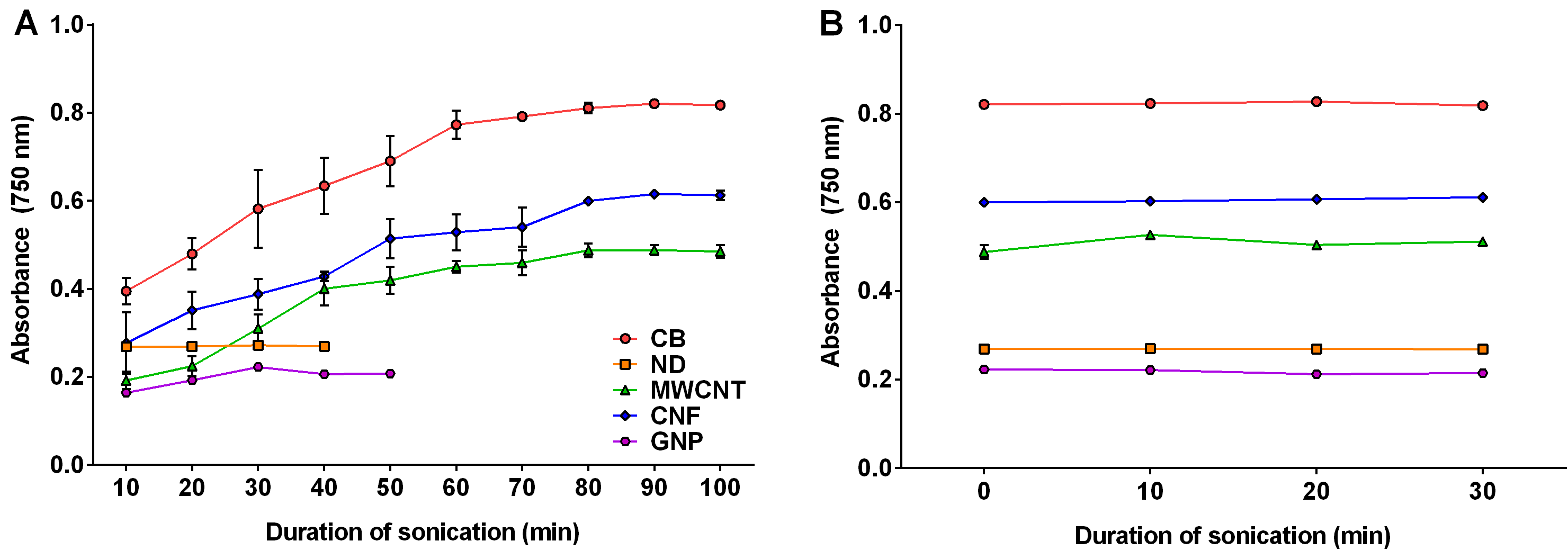


**Figure S2**. Evaluation of the dispersibility of CNMs. The time-course dispersibility of stock solution (A) and working solution (B) of CNMs. (A), To evaluate the dispersibility of the stock solution, 1 mg/mL stock solution was sonicated for 10 min – 100 min. Then, at each time-point, the stock solution was diluted in DW at 25 µg/mL with vigorous vortexing for 30 sec and measured the optical density at 750 nm. (B), To evaluate the duration of sonication for working solution, the working solution (25 µg/mL) of each NM after an optimal sonication duration of stock solution (see Table 4) was sonicated further up to 30 min and optical density was measured at 750 nm. *n*=4.


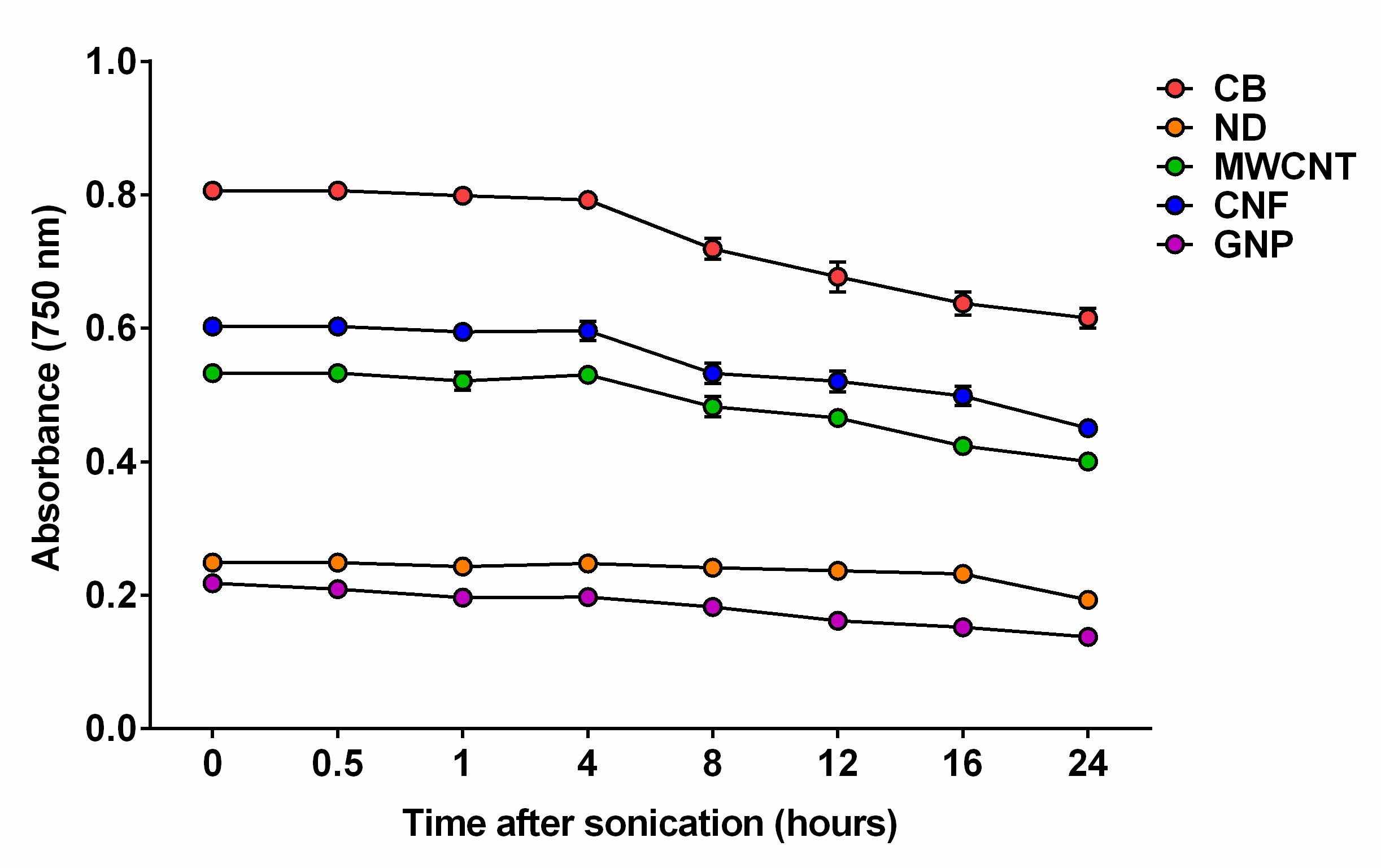


**Figure S3**. Duration of the dispersion stability of CNMs. The working solution of CNMs at 25 µg/mL was sonicated for 10 min after an optimal sonication duration of stock solution (see Table 4). Then, the duration of the dispersion stability was measured at each time-point up to 24 h. *n*=4.

Table S1. The recovery rates of CB, MWCNT, and CNF from lung tissue homogenates following proteinase K digestion with quantification using the UV-Vis spectrophotometer technique.

| CNMs | Target concentration (μg/mL) | | | | | | | | Overall |
| --- | --- | --- | --- | --- | --- | --- | --- | --- | --- |
|  | 3.1 | 4.7 | 6.2 | 9.4 | 12.5 | 18.7 | 25 | 37.5 |  |
| CB | | | | | | | | |  |
| Measured | 2.96 ± 0.05 | 4.51 ± 0.06 | 6.00 ± 0.08 | 8.97 ± 0.07 | 11.97 ± 0.17 | 17.98 ± 0.11 | 24.14 ± 0.24 | 35.85 ± 0.70 |  |
| Recovery % | 95.0 | 95.9 | 96.0 | 95.4 | 95.8 | 96.1 | 96.5 | 95.6 | 95.8 |
| MWCNT | | | | | | | | |  |
| Measured | 2.92 ± 0.04 | 4.47 ± 0.08 | 5.98 ± 0.08 | 8.87 ± 0.04 | 11.93 ± 0.20 | 17.73 ± 0.32 | 24.54 ± 0.20 | 35.83 ± 0.68 |  |
| Recovery % | 93.6 | 95.2 | 95.6 | 94.4 | 95.5 | 94.8 | 98.1 | 95.5 | 95.3 |
| CNF | | | | | | | | |  |
| Measured | 2.91 ± 0.03 | 4.50 ± 0.06 | 5.95 ± 0.08 | 8.99 ± 0.08 | 11.97 ± 0.17 | 17.87±0.10 | 24.48±0.05 | 35.96±0.61 |  |
| Recovery % | 93.5 | 95.8 | 95.3 | 95.6 | 95.8 | 95.6 | 97.9 | 95.9 | 95.7 |

Table S2. The recovery rates of ND and GNP from lung tissue homogenates following proteinase K digestion with quantification using the UV-Vis spectrophotometer technique.

| CNMs | Target concentration (μg/mL) | | | | | | | | Overall |
| --- | --- | --- | --- | --- | --- | --- | --- | --- | --- |
|  | 9.4 | 12.5 | 18.7 | 25 | 37.5 | 50 | 75 | 100 |  |
| ND | | | | | | | | |  |
| Measured | 8.19 ± 0.16 | 11.24 ± 0.25 | 16.15 ± 0.61 | 22.43 ± 0.95 | 33.59 ± 1.65 | 47.58 ± 1.96 | 68.22 ± 3.00 | 95.10 ± 4.20 |  |
| Recovery % | 87.1 | 89.9 | 86.4 | 89.7 | 89.5 | 95.1 | 90.9 | 95.1 | 90.5 |
| GNP | | | | | | | | |  |
| Measured | 8.31 ± 0.14 | 10.92 ± 0.28 | 16.89 ± 0.48 | 24.16 ± 0.77 | 36.18 ± 0.71 | 48.26 ± 1.22 | 72.91 ± 2.42 | 97.64 ± 3.59 |  |
| Recovery % | 88.5 | 87.4 | 90.3 | 96.6 | 96.4 | 96.5 | 97.2 | 97.6 | 93.8 |

Table S3. The screening result of NIR absorbance at 750 nm of various types of nanomaterials.

| Dose (μg/mL) | CB | CoO | Co_3_O_4_ | CuO | NiO | SiO_2_ | TiO_2_ | ZnO |
| --- | --- | --- | --- | --- | --- | --- | --- | --- |
| 0.39 | 0.0139* | 0.0088 | 0.0083 | 0.0097 | 0.0085 | 0.0084 | 0.0082 | 0.0084 |
| 0.78 | 0.0264 | 0.0092 | 0.0087 | 0.0083 | 0.0087 | 0.0083 | 0.0097 | 0.0087 |
| 1.56 | 0.0527 | 0.0087 | 0.0089 | 0.0086 | 0.0082 | 0.0080 | 0.0132* | 0.0087 |
| 3.12 | 0.1029 | 0.0104* | 0.0101* | 0.0085 | 0.0079 | 0.0085 | 0.0253 | 0.0092* |
| 6.25 | 0.2052 | 0.0202 | 0.0199 | 0.0085 | 0.0090* | 0.0097 | 0.0493 | 0.0182 |
| 12.5 | 0.4103 | 0.0418 | 0.0447 | 0.0103* | 0.0161 | 0.0102* | 0.0902 | 0.0374 |
| 25 | 0.8170 | 0.0847 | 0.0841 | 0.0206 | 0.0321 | 0.0202 | 0.1721 | 0.0770 |
| 50 | 1.6557 | 0.1786 | 0.1698 | 0.0412 | 0.0651 | 0.0415 | 0.3368 | 0.1505 |
| 100 | 1.8821 | 0.3771 | 0.3371 | 0.0825 | 0.1297 | 0.0832 | 0.6268 | 0.3034 |
| 200 | 1.9825 | 0.7399 | 0.6698 | 0.1650 | 0.2594 | 0.1634 | 1.1481 | 0.6043 |
| 400 | 2.0887 | 1.3888 | 1.3064 | 0.3306 | 0.5192 | 0.3275 | 1.9762 | 1.1548 |
| 600 | 2.1675 | 1.8010 | 1.7770 | 0.5044 | 0.7770 | 0.4913 | 2.0765 | 1.8519 |
| 800 | 2.1156 | 1.9824 | 2.0633 | 0.6572 | 1.0926 | 0.6549 | 2.0427 | 1.9966 |
| 1000 | 2.1085 | 2.0015 | 2.1235 | 0.8225 | 1.3006 | 0.8187 | 2.0758 | 2.0351 |

CoO, Co_3_O_4_, NiO, SiO_2_, and TiO_2_ were purchased from NanoAmor (Houston, TX, USA); CB was purchased from Evonik Degussa GmbH (Frankfurt, Germany); CuO was purchased from Sigma-Aldrich (St. Louis, MO, USA); ZnO was purchased from NanoScale Corporation (Manhattan, KS, USA).

*The lowest measurable concentration. *n*=4 for each dose group.
